# A biogeographic perspective on the evolution of fire syndromes in pine trees (*Pinus*: Pinaceae)

**DOI:** 10.1098/rsos.172412

**Published:** 2018-03-21

**Authors:** Kevin J. Badik, Joshua P. Jahner, Joseph S. Wilson

**Affiliations:** 1 The Nature Conservancy, 1 East First Street, Suite 1007, Reno, NV 89501, USA; 2 Department of Biology, University of Nevada, Reno, NV 89557, USA; 3 Department of Biology, Utah State University, Tooele, UT 84074, USA

**Keywords:** Beringia, biogeography, evolution, fire, trait reconstruction, parallel evolution

## Abstract

Our goals were to explore the relationship between biogeography and the evolution of fire-adaptive syndromes in the genus *Pinus*. We used a previously published time-calibrated phylogeny and conducted ancestral trait reconstruction to estimate the likely timing of diversification in *Pinus*, and to determine when fire-adaptive syndromes evolved in the lineage. To explore trait conservation among fire syndromes and to investigate historical biogeography, we constructed ancestral state reconstructions using the program RASP and estimated the degree of conservatism for fire-adapted traits in the program BaTS. Our reconstructions suggest that the Bering land bridge, which connected North America and Asia, probably played a major role in early pine evolution. Our estimates indicated that fire-adaptive syndromes seem to have evolved more frequently in New World taxa and probably are related to the uplift of major North American mountain ranges. Our data suggest that certain geographically widespread adaptations to fire evolved repeatedly, possibly due to localized changes in climate and environment, rather than resulting from large dispersal events of pre-adapted individuals.

## 1. Introduction

Understanding the origin and distribution of ecological traits within related taxa is an important goal in evolutionary biology (e.g. [1,2]). Some characters evolve repeatedly under similar selection pressures (parallel evolution), such as C_4_ photosynthesis in grasses [3] or mimicry rings in New World velvet ants [4], while others have a single common ancestor, such as the presence of arbuscular mycorrhiza among plants [5]. Additionally, the distribution of characters can be clustered across a phylogeny (e.g. [6]), resulting from closely related species sharing more similar traits than distant relatives (i.e. phylogenetic signal [7]). While many studies of trait evolution often focus on a single trait of interest, characters can also evolve together as a suite of correlated traits, such as pollination [8] and seed syndromes [9]. To fully understand how traits and/or associated syndromes evolve in a particular lineage requires the examination of phylogenetic, biogeographic and ecological data [10]. Additionally, understanding the biogeography and evolution of a particular taxon can aid in understanding how it may evolve in the future as environmental conditions shift.

Fire is an important ecological process in many natural systems and has been a component of plant communities around the world since at least the Silurian period (approx. 443.7–416 Ma) [11]. Fire has probably acted as a selective force for a wide range of plants, including members of the families Poaceae, Proteaceae, Ericaceae and Fagaceae [12]. Fire-prone habitats, such as those found in Mediterranean climate zones, savannahs and dry forests, contain species that have developed traits to cope with the presence of fire on the landscape. Fire-adapted traits have independently arisen across a wide range of plant taxa in both angiosperm and gymnosperm lineages [6]. These traits include re-sprouting (both epicormically and from below ground), thick bark to insulate against heat, shedding of low canopy branches, serotiny and smoke-induced germination [13–15]. Researchers have noted that these traits tend to be grouped in different ecologically significant strategies or syndromes (see additional description of fire syndromes below) [16–18] and that these fire-adapted traits may be evolutionarily conserved within lineages [19,20]. The optimal fire syndrome for a species is related to the frequency and severity of the fire regime in a given environment [21], and is affected by evolutionary history [22].

Pines (*Pinus* L.) are a dominant forest component in much of the Northern Hemisphere and have been naturalized in the Southern Hemisphere [23,24]. In addition to being a cosmopolitan genus, pines inhabit a wide range of habitat ranging from arid Mediterranean climates to subtropical forests [25] across two subgenera, *Pinus* L. and *Strobus* Lemmon. Generally speaking, members of subgenus *Pinus* often occupy more productive and fire prone habitats, while those in subgenus *Strobus* inhabit less productive, more stressful environments (e.g. deserts and alpine habitats) [15]. In similar environments, distantly related pines often share similar ecological traits [17], consistent with parallel evolution. Because pines are the dominant species in many fire-prone systems [15], much attention has been given to the relationship of the genus with fire, both ecologically and evolutionarily (e.g. [18,24,26–28]). The distribution of pines across the Northern Hemisphere enables us to examine what role historical biogeography plays in the evolution of ecological traits within the genus.

Reconstructions of the ancestral trait condition or historical biogeography have been conducted for a variety of taxa [1,29,30] including for the genus *Pinus* [31,32]. However, fewer studies have compared the historical biogeography and ancestral trait conditions within the same study, though it is informative to relate the two processes (e.g. [24,33]). Most historical biogeography reconstructions of *Pinus* have been large scale, discerning between Old World and New World taxa [31]. Regional-level reconstructions might be more appropriate, as several areas have been identified as important centres of *Pinus* diversity (e.g. eastern North America and Central America) [34]. Additionally, regional-level studies allow for the integration of diversification patterns with geologic changes that may be missed at a larger scale, allowing a link to be made between dispersal history and the evolution of ecologically significant traits. We take this approach a step further by using a more detailed regional map based on current species richness to describe the link between historical biogeography and the evolution of fire syndromes across *Pinus*.

Here, we present analyses that explore the historical biogeography of *Pinus,* addressing how geologic history might have shaped the evolution of fire syndromes within the genus. We used a combination of ancestral trait reconstruction and historical biogeography to address the following questions: (1) Did specific fire syndromes evolve repeatedly throughout the diversification of *Pinus* or were they derived from single common ancestors? (2) What are the likely geographical origins of both the genus (*Pinus*) and the subgenera (*Pinus* and *Strobus*)? (3) Can our reconstructions of ancestral areas and transcontinental dispersal events be linked to any major geologic events? (4) Do the two subgenera, *Pinus* and *Strobus*, share a common or divergent biogeographic history, i.e. did the same historical events lead to diversification in both groups?

## 2. Material and methods

Morphological and life-history traits described by He *et al.* [32] were used to designate the fire syndrome for all 113 *Pinus* species included in the analysis (electronic supplementary material, table S1; additionally morphological information was gathered from the Gymnosperm database [35]). We used the terminology put forth by Rowe [16] (though see [14] for a similar classification), which separates plants’ response to fire into five groups based on life histories. *Invaders* are highly dispersed individuals with rapid growth rates and tend to be early seral species; none of the pines were placed in this category. *Resisters* are species where adults tend to survive surface fires, typically due to thick bark and dropping of low hanging fuels, though immature individuals can face high mortality (e.g. Jeffrey pine; *P. jeffreyi*). *Evaders* are species where adults are killed during fires (through the consumption of the canopy); however, these individuals have aerial (i.e. serotiny) or soil seed storage that protects seeds from heat and enables their offspring to germinate in the newly burned sites (e.g. Jack pine; *P. banksiana*). *Endurers* are species that survive fire through re-sprouting, either from below-ground structures or epicormically from above-ground structures; these species also generally employ other fire-adaptive strategies. For example, slash pine (*P. elliottii*) has both resister and endurer strategies, meaning it has adaptations to resist fires, but is also capable of re-sprouting after a fire. Lastly, *avoiders* are individuals that lack traits that allow an individual or its offspring to persist following a fire event (e.g. bristlecone pine; *P. longaeva*). These species tend to inhabit environments where fires are unlikely, such as low productivity sites. As it is possible for a species to possess multiple fire-adapted traits (e.g. Canary Island pine; *P. canariensis*), several pines were placed into multiple categories, though avoiders were never jointly categorized with any of the other three strategy types.

**Figure 1.**
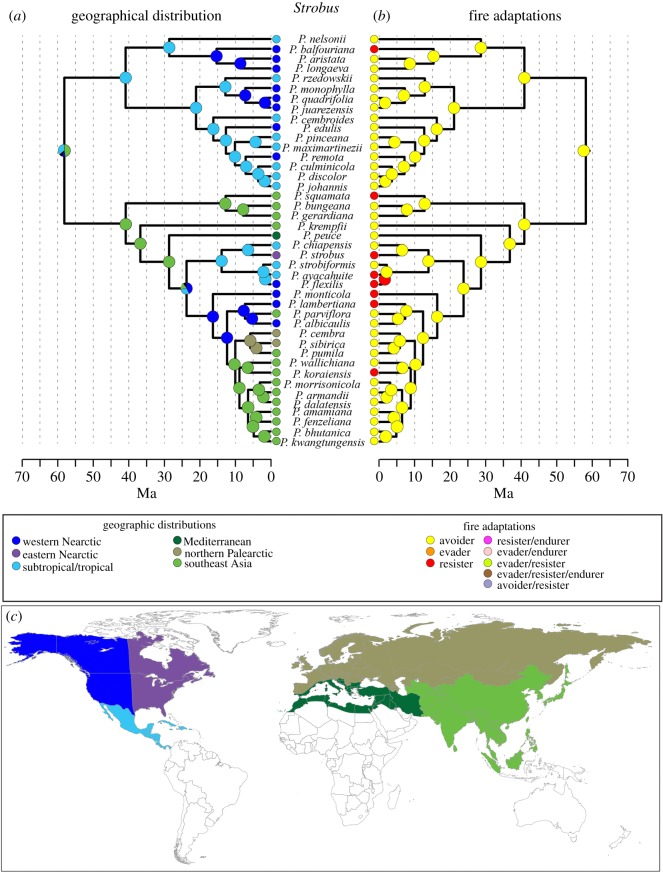
The phylogenetic tree for subg. *Strobus*. Panel (*a*) represents the reconstructed ancestral locations; (*b*) represents the reconstructed ancestral fire syndromes. Solid coloured circles represent nodes where Bayesian posterior probability for ancestral location or fire syndrome was greater than 0.5. Panel (*c*) is a map of the regions that were used for the geographical distribution.

Contemporary range data were collected for North American, European and Asian species [35], Mexican and Central American species [36], and Mediterranean species [26] (electronic supplementary material, table S1). Six geographical regions were designated (figure 1*c*). These regions were selected based on patterns of extant pine ranges and species diversity, as well as ecological differences in fire regimes among the regions.

To explore the geographical distributional history as well as the evolution of fire adaptation in *Pinus*, we used ancestral trait reconstruction to infer both the likely ancestral geographical centres of origin and likely ancestral fire syndrome. These analyses estimate the likelihood of each potential character state (here fire syndrome or centre of origin) for each ancestral node in a phylogeny based on the characteristics or distributions of extant species (i.e. the states of extinct species are not considered). We used the *Pinus* phylogeny proposed by Gallien *et al.* [24], which was reconstructed using eight plastid gene regions and time-calibrated using four fossils. While He *et al.* [32] reconstructed ancestral traits for individual fire-adapted traits, and Gallien *et al.* [24] investigated the historical biogeography, here we investigate how historical biogeography and the evolution of fire syndromes may be interconnected. Fire syndromes and current geographical locations were mapped onto two separate trees, one tree for each subgenus. For each trait (fire syndrome and geographical location), trait reconstruction was performed using rasp v.2.0b (Reconstruct Ancestral State in Phylogenies [37]) with the Bayesian binary MCMC analysis (BBM). BBM uses a hierarchical Bayesian model to infer states at ancestral nodes in a phylogeny based on traits from extant species. In the BBM analysis, state frequency transitions can be modelled with a fixed frequency (JC model) or a variable frequency (F81 model). The F81 model of evolution was implemented in each analysis and different rates of change among ancestral states were allowed to reduce any constraints on the reconstruction of ancestral states. Default values were selected for all other MCMC parameters.

To investigate the degree of phylogenetic conservatism found in each fire syndrome individually, the maximum monophyletic clade (MC) size [38] was calculated for each syndrome and compared to the distribution of MC values from 1000 trees with randomized trait values. MC values were calculated and compared with the Bayesian Tip-association Significance Testing (BaTS) software [38]. The BaTS analysis allows for the examination of multiple traits within the same analysis.

## 3. Results

**Figure 2.**
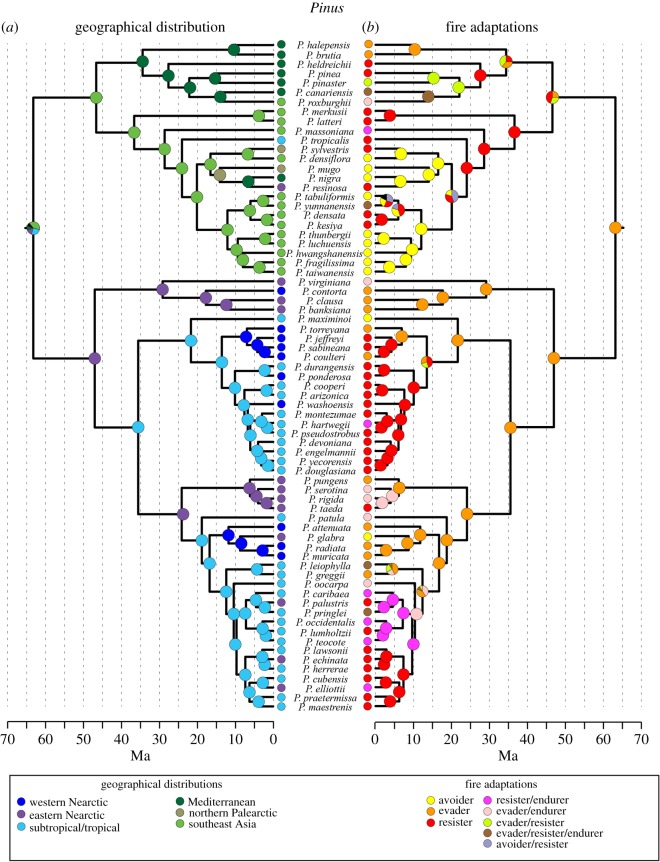
The phylogenetic tree for subg. *Pinus*. Panel (*a*) represents the reconstructed ancestral locations; (*b*) represents the reconstructed ancestral fire syndromes. Solid coloured circles represent nodes where Bayesian posterior probability for ancestral location or fire syndrome was greater than 0.5.

Among the 113 species in the Gallien *et al.* [24] phylogeny, the most common fire strategy was the avoider strategy (table 1). Within fire-adaptive syndromes, the resister strategy was over twice as common as any other fire-adapted strategy (table 1). When the levels of niche conservatism were estimated individually for the different syndromes in the MC analysis, the avoider and evader syndromes were significantly conserved, while the resister strategy appears randomly throughout the lineage (table 1), having evolved independently on multiple occasions (figures 1*b* and 2*b*).

**Table 1.** The estimates of trait conservatism for fire syndromes within genus *Pinus* from the BaTS analysis. Significance indicates a non-random distribution of the syndrome (i.e. a conserved niche). Significant values are indicated in italics. ‘MC’ is the maximum monophyletic clade.

trait	*N*	observed MC	null MC	*p*-value
avoider	45	12	3.084	<*0*.*001*
evader	12	3	1.352	*0*.*016*
resister	39	4	2.778	0.172
evader, endurer	6	1	1.087	1.000
evader, resister	1	1	1.000	1.000
resister, endurer	6	1	1.094	1.000
evader, resister, endurer	4	1	1.024	1.000

While the node is not included in the figures, our results do not clearly delineate the geographical location for the ancestor for the genus *Pinus*, though the Bayesian posterior probabilities (Bpp) indicate that southeast Asia has the highest support (49% Bpp) followed by subtropical North America (30% Bpp) and eastern North America (8% Bpp), with unresolved areas contributing to the remainder (13% Bpp). Similarly, the subg. *Strobus* is most likely to have either a southeast Asian ancestor (47% Bpp, figure 1*a*) or a subtropical Nearctic ancestor (32% Bpp, figure 1*a*). The ancestor of subg. *Pinus* was less resolved with ancestral areas predicted to be southeast Asian (42% Bpp, figure 2*a*), Nearctic (subtropical 25% Bpp, eastern Nearctic 15%, figure 2*a*) or Mediterranean (15% Bpp, figure 2*a*). Our data suggest ancestral pines in both subgenera migrated between Asia and North America starting around 65–40 Ma (figures 1 and 2). In *Strobus,* there is evidence for further migration between Asia and North America at around 25 Ma and again at around 10 Ma (figure 1*a*). Alternatively, in *Pinus* there is one migration event between Asia and North America between 65 Ma and 45 Ma, but no major additional migrations with the exception of two New World *Pinus* species, *P. tropicalis* and *P. resinosa*, which are both found in a large Old World clade (figure 2*a*).

Western North America was only invaded five times, three times in *Strobus* and two times in *Pinus*. Interestingly, these movements into western North America all occurred after about 20 Ma, which closely corresponds to estimated ages of major mountain-building events and aridification [39]. Another interesting pattern in these western invasions is that *Pinus* invaders colonized the fire-adapted low-elevation niches, while S*trobus* invaders took the high elevation, less fire-prone areas (figures 1 and 2).

In *Strobus*, which contains mainly non-fire-adapted species, the only fire syndrome to evolve is the resister strategy (figure 1*b*). Of the eight fire-adapted species, only two are found outside of the New World regions. Furthermore, in *Strobus* fire adaptations did not evolve until relatively recently (during or after the Pleistocene). In *Pinus*, fire adaptions are found throughout the tree, with three reversals from fire-adapted ancestors to species lacking fire adaptations (figure 2*b*). The majority of *Pinus* species that have lost fire adaptations are found in southeast Asia (figure 2).

The analyses of ancestral fire syndromes indicate that the *Pinus* ancestor exhibited avoider traits (71% Bpp: node not shown on the phylogeny). Our results also indicate that ancestral subg. *Strubus* species were avoiders (i.e. not adapted to fire). The ancestral subg. *Pinus* species, however, were probably fire-adapted using evader strategies. The evolutionary event leading to the evader and avoider ancestors occurred around 65 Ma and probably happened in southeast Asia (figures 1 and 2).

## 4. Discussion

Our results show that *Pinus* has a complex evolutionary and geographical history, with migration occurring between North America and Eurasia multiple times. Like previous studies [24,31], our analysis supports a Eurasian origin for pines (figures 1 and 2), though only with weak support. Additionally, across the genus, pines show both niche conservatism (especially with the evader and avoider syndromes) and flexibility to adapt to changing conditions, suggested by the multiple independent evolution events of different fire syndromes, especially the resister strategy.

Some divergence events appear to have been driven by ecological rather than geographical events. For example, the split between the *Pinus* and *Strobus* appears to be related to the evolution of fire adaptations in *Pinus* but not in *Strobus* (figures 1 and 2). There is little evidence in our analysis that vicariant events played a role in this divergence. Similar results have been found in other plant groups showing Asian and North American connections, where taxa were thought to have diversified in Asia prior to moving to North America [40]. The estimated age for this early divergence in pines is predicted to be in the Paleocene/Eocene boundary, around 55 Ma (figures 1 and 2). This time period, which is often called the Paleocene–Eocene Thermal Maximum (PETM), is thought to have been one of the warmest periods of the Cenozoic with little or no polar ice and global surface temperatures much warmer than at present [41]. The PETM has been linked to diversification and global expansion of a variety of taxa (e.g. [40,42]). We suggest Beringia probably led to the dispersal from Asia to North America of ancestral pine species during the PETM as has been found for other taxa [43,44].

We not only find a Paleogene connection between Asia and North America, but we also find evidence of migration and diversification across the Bering land bridge in the Oligocene/Miocene (figure 1*a*). Similar geographical patterns linked to these dates have also been found in a variety of other organisms [40,45]. In fact, the Bering land bridge has led to multiple dispersal events between Asia and North America over the past 65 Ma [46].

Interestingly, the historical biogeography of *Strobus* shows multiple dispersals across the Bering land bridge, yet *Pinus* largely remains separated on the two continents after the original migration (figures 1 and 2). This might be related to the fire adaptations that evolved in the two groups. *Strobus,* for instance, is largely made up of avoiders, while *Pinus* has evolved a variety of different fire adaptations. It is possible that the evolution of fire adaptations resulted in taxa that were less capable of continental scale migration, as we see little evidence of fire-adapted ancestors moving between continents, yet we see multiple instances of non-fire-adapted ancestors moving between Asia and North America (figures 1 and 2).

Multiple connections between Old and New World plant groups have been found through phylogeographic analyses [40]. These biogeographic connections can be lumped into two main categories, Pacific track migration and Atlantic track migration [40]. Atlantic track events are those that connected ancestral plant taxa between European and North American regions via land bridges through Greenland, while Pacific track migrations connected Asia to North America via the Bering land bridge [40]. While several biologic connections have been found between North America and Europe [40,42], in pines we only find that the Pacific track of migration between Old and New World areas is supported.

Most of the historical invasions of pines into North America are estimated to be into either subtropical areas or eastern North America (figures 1 and 2). While this may be surprising, particularly because ancestral species would have to move through western North America to get to either subtropical or eastern areas, Neogene orogenic mountain building events in western North America dramatically changed the climate and ecology of the region [39]. These changes probably pushed out some taxa and allowed the evolution of new western North American species, which our analyses suggest happened beginning in the Early Miocene (figures 1 and 2).

Near the Paleocene/Eocene boundary was when our reconstruction inferred the development of fire syndromes in pines (figure 2). The ancestor of subg. *Pinus* was inferred to be an evader, while the ancestor of subg. *Strobus* was reconstructed to be an avoider. The development of fire syndromes during the Early Eocene is of note considering the hypothesized climate conditions during the Eocene. Evidence suggests that during the Cretaceous period fires were relatively frequent [47,48] due to high oxygen atmospheric content [49]. Global fire activity appears to have declined following the Cretaceous period, accompanied by the expansion of angiosperm-dominated rainforests. These less flammable forests may have led to reduced fire activity [50]. Therefore, the Eocene would appear to be an unlikely time for fire syndromes to develop. However, the Eocene was also characterized by fluctuating climates [51]. Additionally, localized concentrations of fossilized charcoal during portions of a short warming period (i.e. PETM) suggest that oxygen concentrations were near current conditions or warmer, and fire was not excluded from some landscapes [52]. Changing global climate coupled with mountain-building events would have localized climates, some of which may have been more suitable for fire and thus led to the evolution of fire syndromes in particular regions.

Our results also indicate that the Miocene was an important period for pine evolution. Members of subg. *Pinus* appeared to make several movements around North America. Mountain-building events during the Miocene and into the Pliocene [39,53], increased seasonality [54] and shifting plant communities have been hypothesized in North America during the Miocene [55]. Evidence suggests an increase in aridity and that fires were more prevalent during the Miocene in western North America, and this increased fire activity may explain the increase in C4 plant distribution [54]. The lack of fire adaptations among subg. *Strobus* would have relegated *Strobus* species to more physiologically stressful, fire-free sites, such as those found at higher elevations.

Within Eurasia, several important events occurred during the Miocene, including further dispersal and radiation in the Mediterranean basin, diversification of avoider-type members of subg. *Pinus* in Asia, and diversification of subg. *Strobus* members in Asia. The Mediterranean pines are noteworthy, as the six closely related species display a variety of fire syndromes (two members exhibit multiple syndromes). These species have not diverged since the Miocene. The radiation of Asian pines of both subgenera comprise mostly taxa that exhibit the avoider strategy. This is despite climatic conditions that would suggest increased fire activity within Asia [54]. The lack of fire-adapted syndromes among Asian pines during the Miocene may reflect the importance of evolutionary constraints. In other words, it could be that while increased fire activity should have led to the evolution of fire adaptations, the Asian taxa may not have had the underlying genetic variation to allow for the development of these strategies.

## 5. Conclusion

Our results provide more details on the biogeography of *Pinus* evolution and the evolutionary relationship pines have with fire. We provide support to previous hypotheses regarding the early development of fire-adapted traits and the importance of the Eocene and Miocene for pine evolution, while providing additional information about the role of geologic events in pine evolution and biogeography. Some recent phylogenetic reconstructions of the genus *Pinus* have estimated slightly different dates of various divergence events within the genus (e.g. [32,56,57]). However, given the lack of consensus in the timing of historical geologic and climatic events [39], our results still provide a complementary perspective on how the historical biogeography of *Pinus* may have influenced the evolutionary ecology of the group. Given the ecological and economic importance of pines, understanding their evolutionary history and biogeography can inform us on how pines might respond to the current changing world. Our phylogenetic evidence suggests that subg. *Strobus* and those members of subg. *Pinus* that possess an evader strategy may possess less evolutionary flexibility, given the level of niche conservatism observed. However, other members of the subg. *Pinus* appear to be more flexible in their response to fire and exhibiting a range of fire syndromes.

## Supplementary Material

Table S1. List of species involved in the analysis with their geographic distribution and their fire adaptations.

## Data accessibility

The datasets supporting this article have been included in the electronic supplementary material.
