# Supplementary material for: A biogeographic perspective on the evolution of fire syndromes in pine trees (Pinus: Pinaceae)
Source: R Soc Open Sci. 2018 Mar 21;5(3):172412. doi: 10.1098/rsos.172412 (PMC5882747; doi:10.1098/rsos.172412)
Supplement: Table S1. List of species involved in the analysis with their geographic distribution and their fire adaptations. [file rsos172412supp1.docx]

| **Table S1.** List of species involved in the analysis with their geographic distribution and their fire adaptations. | | |
| --- | --- | --- |
| **Species** | **Geography** | **Fire adaptation** |
| *Pinus albicaulis* | Western Nearctic | Avoider |
| *Pinus amamiana* | Southeast Asia | Avoider |
| *Pinus aristata* | Western Nearctic | Avoider |
| *Pinus arizonica* | Neo-Subtropical/tropical | Resister |
| *Pinus armandii* | Southeast Asia | Avoider |
| *Pinus attenuata* | Western Nearctic | Evader |
| *Pinus ayacahuite* | Neo-Subtropical/tropical | Resister |
| *Pinus balfouriana* | Western Nearctic | Resister |
| *Pinus banksiana* | Eastern Nearctic | Evader |
| *Pinus bhutanica* | Southeast Asia | Avoider |
| *Pinus brutia* | Mediterranean | Evader |
| *Pinus bungeana* | Southeast Asia | Avoider |
| *Pinus canariensis* | Mediterranean | Evader, Endurer, Resister |
| *Pinus caribaea* | Neo-Subtropical/tropical | Resister, Endurer |
| *Pinus cembra* | Northern Palearctic | Avoider |
| *Pinus cembroides* | Neo-Subtropical/tropical | Avoider |
| *Pinus chiapensis* | Neo-Subtropical/tropical | Avoider |
| *Pinus clausa* | Eastern Nearctic | Evader |
| *Pinus contorta* | Western Nearctic | Evader |
| *Pinus cooperi* | Neo-Subtropical/tropical | Resister |
| *Pinus coulteri* | Western Nearctic | Evader |
| *Pinus cubensis* | Neo-Subtropical/tropical | Resister |
| *Pinus culminicola* | Neo-Subtropical/tropical | Avoider |
| *Pinus dalatensis* | Southeast Asia | Avoider |
| *Pinus densata* | Southeast Asia | Resister |
| *Pinus densiflora* | Southeast Asia | Avoider |
| *Pinus devoniana* | Neo-Subtropical/tropical | Resister |
| *Pinus discolor* | Neo-Subtropical/tropical | Avoider |
| *Pinus douglasiana* | Neo-Subtropical/tropical | Resister |
| *Pinus durangensis* | Neo-Subtropical/tropical | Resister |
| *Pinus echinata* | Eastern Nearctic | Resister |
| *Pinus edulis* | Western Nearctic | Avoider |
| *Pinus elliottii* | Eastern Nearctic | Resister, Endurer |
| *Pinus engelmannii* | Neo-Subtropical/tropical | Resister |
| *Pinus fenzeliana* | Southeast Asia | Avoider |
| *Pinus flexilis* | Western Nearctic | Resister |
| *Pinus fragilissima* | Southeast Asia | Avoider |
| *Pinus gerardiana* | Southeast Asia | Avoider |
| *Pinus glabra* | Eastern Nearctic | Avoider |
| *Pinus greggii* | Neo-Subtropical/tropical | Evader |
| *Pinus halepensis* | Mediterranean | Evader |
| *Pinus hartwegii* | Neo-Subtropical/tropical | Resister, Endurer |
| *Pinus heldreichii* | Mediterranean | Resister |
| *Pinus herrerae* | Neo-Subtropical/tropical | Resister |
| *Pinus hwangshanensis* | Southeast Asia | Avoider |
| *Pinus jeffreyi* | Western Nearctic | Resister |
| *Pinus johannis* | Neo-Subtropical/tropical | Avoider |
| *Pinus juarezensis* | Western Nearctic | Avoider |
| *Pinus kesiya* | Southeast Asia | Resister |
| *Pinus koraiensis* | Southeast Asia | Resister |
| *Pinus krempfii* | Southeast Asia | Avoider |
| *Pinus kwangtungensis* | Southeast Asia | Avoider |
| *Pinus lambertiana* | Western Nearctic | Resister |
| *Pinus latteri* | Southeast Asia | Resister |
| *Pinus lawsonii* | Neo-Subtropical/tropical | Resister |
| *Pinus leiophylla* | Neo-Subtropical/tropical | Resister, Endurer, Evader |
| *Pinus longaeva* | Western Nearctic | Avoider |
| *Pinus luchuensis* | Southeast Asia | Avoider |
| *Pinus lumholtzii* | Neo-Subtropical/tropical | Resister |
| *Pinus maestrensis* | Neo-Subtropical/tropical | Resister |
| *Pinus massoniana* | Southeast Asia | Resister, Endurer |
| *Pinus maximartinezii* | Neo-Subtropical/tropical | Avoider |
| *Pinus maximinoi* | Neo-Subtropical/tropical | Avoider |
| *Pinus merkusii* | Southeast Asia | Resister |
| *Pinus monophylla* | Western Nearctic | Avoider |
| *Pinus montezumae* | Neo-Subtropical/tropical | Resister |
| *Pinus monticola* | Western Nearctic | Resister |
| *Pinus morrisonicola* | Southeast Asia | Avoider |
| *Pinus mugo* | Northern Palearctic | Avoider |
| *Pinus muricata* | Western Nearctic | Evader |
| *Pinus nelsonii* | Neo-Subtropical/tropical | Avoider |
| *Pinus nigra* | Mediterranean | Avoider |
| *Pinus occidentalis* | Neo-Subtropical/tropical | Resister, Endurer |
| *Pinus oocarpa* | Neo-Subtropical/tropical | Evader, Endurer |
| *Pinus palustris* | Eastern Nearctic | Resister |
| *Pinus parviflora* | Southeast Asia | Avoider |
| *Pinus patula* | Neo-Subtropical/tropical | Evader, Endurer |
| *Pinus peuce* | Mediterranean | Avoider |
| *Pinus pinaster* | Mediterranean | Evader, Resister |
| *Pinus pinceana* | Neo-Subtropical/tropical | Avoider |
| *Pinus pinea* | Mediterranean | Resister |
| *Pinus ponderosa* | Western Nearctic | Resister |
| *Pinus praetermissa* | Neo-Subtropical/tropical | Resister |
| *Pinus pringlei* | Neo-Subtropical/tropical | Evader, Endurer, Resister |
| *Pinus pseudostrobus* | Neo-Subtropical/tropical | Resister |
| *Pinus pumila* | Southeast Asia | Avoider |
| *Pinus pungens* | Eastern Nearctic | Evader |
| *Pinus quadrifolia* | Western Nearctic | Avoider |
| *Pinus radiata* | Western Nearctic | Evader |
| *Pinus remota* | Western Nearctic | Avoider |
| *Pinus resinosa* | Eastern Nearctic | Resister |
| *Pinus rigida* | Eastern Nearctic | Evader, Endurer |
| *Pinus roxburghii* | Southeast Asia | Evader, Endurer |
| *Pinus rzedowskii* | Neo-Subtropical/tropical | Avoider |
| *Pinus sabineana* | Western Nearctic | Resister |
| *Pinus serotina* | Eastern Nearctic | Evader, Endurer |
| *Pinus sibirica* | Northern Palearctic | Avoider |
| *Pinus squamata* | Southeast Asia | Resister |
| *Pinus strobiformis* | Neo-Subtropical/tropical | Avoider |
| *Pinus strobus* | Eastern Nearctic | Resister |
| *Pinus sylvestris* | Northern Palearctic | Resister |
| *Pinus tabuliformis* | Southeast Asia | Avoider |
| *Pinus taeda* | Eastern Nearctic | Resister |
| *Pinus taiwanensis* | Southeast Asia | Avoider |
| *Pinus teocote* | Neo-Subtropical/tropical | Resister, Endurer |
| *Pinus thunbergii* | Southeast Asia | Avoider |
| *Pinus torreyana* | Western Nearctic | Evader |
| *Pinus tropicalis* | Neo-Subtropical/tropical | Resister |
| *Pinus uncinata* | Northern Palearctic | Avoider |
| *Pinus virginiana* | Eastern Nearctic | Evader, Endurer |
| *Pinus wallichiana* | Southeast Asia | Avoider |
| *Pinus washoensis* | Western Nearctic | Resister |
| *Pinus yecorensis* | Neo-Subtropical/tropical | Resister |
| *Pinus yunnanensis* | Southeast Asia | Evader, Endurer, Resister |
